# Bilaterally isolated abducens palsy after an aneursym rupture is related with intracranial hypertension

**DOI:** 10.1186/s40064-015-1560-z

**Published:** 2015-12-10

**Authors:** Tse-Lun Wang, Chieh-Hsin Wu, Chao-Wen Chen, Tai-Hsin Tsai, Sui-sum Kung, Chia-Hung Chao, Chih-Lung Lin, Yu-Feng Su

**Affiliations:** Department of Neurosurgery, Kaohsiung Medical University Hospital, No 100, Tz-you 1st Road, Kaohsiung, 807 Taiwan; Division of Traumatology, Department of Surgery, Kaohsiung Medical University Hospital, Kaohsiung Medical University, Kaohsiung, Taiwan; Graduate Institute of Clinical Medicine, College of Medicine, Kaohsiung Medical University, Kaohsiung, Taiwan; Graduate Institute of Medicine, College of Medicine, Kaohsiung Medical University, Kaohsiung, Taiwan

**Keywords:** Abducens palsy, Intracranial pressure, Ruptured aneurysm, Subarachnoid hemorrhage, Neuromonitoring

## Abstract

**Background:**

Bilateral and isolated abducens nerve palsy is a rare initial presentation after aneurysms rupture. Several possible mechanisms including intracranial hypertension have been purposed. To date, there have been no reports with objective measurements to demonstrate the relationship between intracranial pressure and isolated abducens palsy in the setting of acute subarachnoid hemorrhage due to aneurysm rupture.

**Findings:**

A 50 year-old female presented with severe headache and bilaterally isolated abducens nerve palsy. A series of image studies showed a ruptured aneurysm over right internal carotid artery and posterior communicating artery bifurcation with minimal subarachnoid hemorrhage. Surgery of aneurysm clipping was performed and intracranial pressure monitoring was applied. Postoperatively no new neurological deficit developed but persistent headache and increased intracranial pressure measured by a fiber-optic device had been observed. The intracranial hypertension then decreased gradually with rapid recovery from the bilateral abducens palsy 7 days after the surgery. The relationship between postoperative intracranial pressure, subarachnoid hematoma and isolated abducens palsy are illustrated.

**Conclusions:**

The report demonstrated the clinical presentation of bilaterally isolated abducens palsy after an intracranial aneurysm rupture is related with the increased intracranial pressure level, rather than the hematoma compression to the nerve or vasospasm of pontine branches of basilar artery.

## Short report

**Fig. 1:**
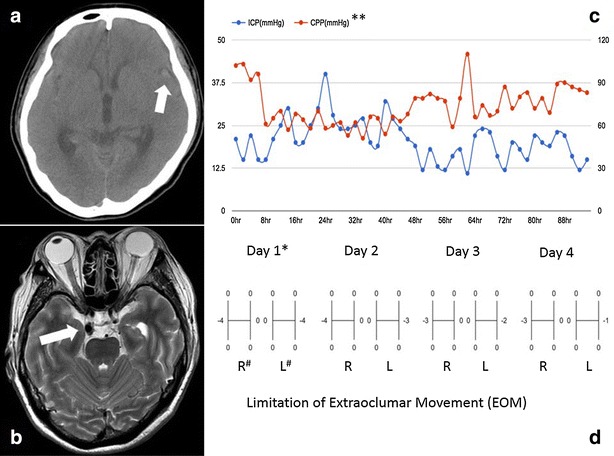
**a** Computed tomography (CT) revealed vague subarachnoid hemorrhage along with left Sylvian fissure (*arrow*) and subdural hematoma over bilateral frontal areas. **b** Magnetic resonance imaging (MRI) showed an aneurysm located in the bifurcation of right internal carotid artery and posterior communicating artery (*arrow*). **c**, **d** The relationship between days after surgery, intracranial pressure, cerebral perfusion pressure and limitation of extraocular movement are illustrated. The lateral gaze limitation of both eyes had a gradually improvement day by day with the decreasing intracranial pressure. **: *ICP* intracranial pressure, *CPP* cerebral perfusion pressure. *: day 1 means day 1 after the surgery. #: “R” means right eye and “L” means “left” side

A 50 year-old female visited our emergency department with sudden onset of severe headache, dizziness, vomiting and diplopia. Recent trauma history was denied. On examination, she was conscious with mild neck stiffness and significantly bilateral abducens palsy. There was no oculomotor neuropalsy. The brain computed tomography (CT) revealed vague hyperdensity along with left Sylvian fissure and bilateral frontal subdural space (Fig. 1a). Minimal subarachnoid hemorrhage and subdural hematoma were considered with undetermined causes. Computed tomography angiography (CTA) was done soon but failed to reveal potential vascular lesions. The magnetic resonance imaging (MRI) and magnetic resonance angiographpy (MRA) then were applied to rule out possible ischemia insult, infection and tumor lesions. However an aneurysm located in the bifurcation of right internal carotid artery and posterior communicating artery (IC-Pcom) was identified (Fig. 1b). The following digital subtraction angiography (DSA) also showed a right IC-Pcom aneurysm with saccular type and measured 1.2 cm in diameter. Surgery of aneurysm clipping was performed early to decrease the chance of re-bleeding. In the operation, brain swelling and high pressure flow were observed during the process of cerebrospinal fluid (CSF) drainage. The brain looked soft and a little sunken later and external ventricular drainage was not applied therefore. The amount of subarachnoid hematoma around the ruptured aneurysm was not much and the aneurysm was clipped smoothly without intraoperative bleeding. Lillequist’s membrane and pre-pontine cistern were not opened. An intraparenchymal pressure monitor (Codman Neuro, MA, USA) was placed over the frontal lobe thereafter.

There was no other new neurological deficit postoperatively. Persistent headache with increased intracranial pressure (IICP), around 30 mmHg, had been noted (Fig. 1c). The figure revealed that the lateral gaze limitation of both eyes had a gradually improvement day by day with the decreasing intracranial pressure (Fig. 1d). Transcranial Doppler was applied to monitor possible vasospasm. Increased velocity of blood flow was observed without other clinical evidences of vasospasm. Seven days after the operation, the patient had a total recovery of the eyes movement.

## Discussion

To the best of our knowledge, this is the first objective measurement report to demonstrate the relationship between IICP and abducens palsy after an intracranial aneurysm rupture.

Ophthamoplegia is one of the clinical presentations of cerebral aneurysms but bilaterally isolated abducens nerve palsy is a rare initial presentation after aneurysms rupture (Koskela et al. 2015). Lesions causing abducens nerve palsy may be located in the brain stem, subarachnoid space, petroclival region, cavernous sinus or in the orbit along the course of the nerve. There have been several possible mechanisms purposed including increased intracranial pressure induced by brain swelling or parenchyma hemorrhage, direct aneurysmal mass effect on abducens nerve, compression by the localization of CSF or hematoma in the basal cistern space, especially in the prepontine cistern (Schneck et al. 2007), and vasospastic pontine branch of basilar artery supplying to abducens nuclei (Ziyal et al. 2003). As the 6th cranial nerve passes through the subarachnoid space, there are three curves in the course of abducens nerve and the most severe curve occurs over the petrous apex. It would be the point most vulnerable to stretch or compression injury, e.g. IICP, brain edema or hematoma (Umansky et al. 1991).

Severe intracranial hypertension immediately after SAH causes inadequate cerebral perfusion and then leads to transient diffuse ischemic encephalopathy (Grote and Hassler 1988). Experimental SAH models have revealed that the initial ischemia results in microcirculatory dysfunction and global cerebral edema may arise very early (Kuyama et al. 1984). Furthermore, progression with diffuse microvascular spams, autoregulatory breakthrough, cerebral inflammation and intracellular shifting of water may precipitate delayed global cerebral edema. Correspondingly global cerebral edema has been shown to be an independent predictor of mortality and poor functional outcome after SAH (Claassen et al. 2002).

For our case, direct aneurysmal mass effect, vasosopastic pontine branch of basilar artery and accumulated hematoma in the cisternal space were all not observed in a series of image studies or intraoperative findings. The high-pressure flow of CSF and brain swelling observed during the surgery revealed intracranial hypertension. Moreover, the postoperative observation showed the trend of IICP decrease and absence of delayed brain edema were compatible with that of abducens palsy recovery (Fig. 1d). The above observation is similar with those in the literature though there was no objective intracranial pressure measurement in the previous reports. The reported recovery period varied from 3 days to 3 months (Ziyal et al. 2003; Nathal et al. 1992; Koskela et al. 2015). Our patient had a rapid and full recovery from the isolated bilateral abducens nerve paralysis by 1 week from onset.

In this case report the CTA failed to show the aneurysm later recognized by MRI and DSA. The possible explanations were technical problems during the procedure or image reconstruction, thrombosed aneurysms and potential vasospasm. Other possible causes of a negative CTA study after diffuse SAH include an extremely small aneurysm, dissection or rupture of an atherosclerotic vessel wall (Agid et al. 2010). The patterns of SAH should be evaluated carefully and further vascular image studies are warranted to rule out underling lesion.

In conclusions, we are convinced that the bilaterally isolated abducens palsy after an intracranial aneurysm rupture in this case is related with the increased intracranial pressure level, rather than the hematoma compression to the nerve or vasospasm of pontine branches of basilar artery.

## Consent to publish

The consent to publish has been obtained from the participant to report individual patient data.

## Abbreviations

CSF: cerebrospinal fluid
CT: computed tomography
CTA: computed tomography angiography
DSA: digital subtraction angiography
IC-Pcom: internal carotid artery and posterior communicating artery
IICP: increased intracranial pressure
MRA: magnetic resonance angiographpy
MRI: magnetic resonance imaging
